# Informal and revolutionary feminist placemaking

**DOI:** 10.3389/fsoc.2024.1347471

**Published:** 2024-02-29

**Authors:** Asma Mehan

**Affiliations:** Huckabee College of Architecture, Texas Tech University, Lubbock, TX, United States

**Keywords:** feminist placemaking, informal placemaking practices, revolutionary placemaking, global south urbanism, Iranian studies

## Abstract

Urban spaces, often emerging outside formal, recognized boundaries, underscore the pivotal role women play in shaping these environments. Despite the enduring influence of patriarchal and hierarchical structures that render these spaces overtly gendered, it is within these contexts that women’s actions become particularly transformative. Drawing from feminist urban theories of the global south, this paper investigates informal placemaking, feminist urban activism, revolutionary placemaking, online protest movements, and the networks that support women’s solidarity groups. Employing a mixed-methods approach that includes case studies, interviews with activists, and social media analysis, this research focuses on Iran, with a specific emphasis on the recent ‘Women, Life, Freedom’ movement. This study not only highlights how women navigate, contest, and reshape urban spaces through feminist urban activism and informal revolutionary placemaking but also anticipates the broader implications of these actions for urban planning and policy. By analyzing and comparing these case studies, we aim to uncover the commonalities, differences, challenges, and opportunities between informal/formal, state-led/bottom-up, and revolutionary feminist placemaking practices in Iran. The findings of this paper are expected to contribute valuable insights into the dynamics of feminist urbanism and suggest avenues for future research in enhancing the inclusivity and responsiveness of urban spaces to gendered needs and activism.

## 1 Introduction

Drawing on the foundational insights of critical sociologists and feminist geographers, this paper examines the gendered dimensions of informal placemaking in Iran. Gender-segregated spaces in Iran represent a dynamic social process influenced by historical, societal, and cultural transformations. This research is anchored in the theoretical frameworks provided by Lefebvre (2009) and McDowell (1999), who have illuminated the intricate ways gender shapes urban spaces. “Informality” in this context is understood as the myriad creative interactions and uses of urban spaces that occur beyond the scope of formal, state-sanctioned activities and regulations. Emphasizing a process-based sociological approach, as advocated by Lamont and Molnar (2002), this paper seeks to describe the properties of generative processes or chains of events that underpin these transformations.

Informality, as conceptualized here, encompasses the unofficial yet normalized practices and structures frequently marginalized or overlooked by formal governance mechanisms. Such informal spaces and processes emerge as vital arenas for transformative actions, particularly in socio-politically challenging contexts (Mehan, 2023a, 2023b). This paper argues that the conventional interpretations of women’s revolutionary placemaking practices found in democratic environments are not directly applicable in non-democratic settings, as suggested by Bayat (2007, p. 160). Bayat (1997) further elaborates that in authoritarian contexts, the traditional avenues for women to organize and mobilize—characterized by collective action, strong leadership, and robust networks—are often curtailed or outright suppressed by the state through a range of tactics from overt violence to surveillance and censorship.

Despite such formidable barriers, the resilience and ingenuity of Iranian women shine through. The case studies presented here illustrate how women employ a variety of informal strategies to circumvent state constraints and advocate for social change. These methods, fraught with challenges and contestation, nonetheless represent critical pathways for resistance and placemaking within Iran’s restrictive political landscape (Mehan, 2024a). Informal revolutionary feminist placemaking, as explored in this paper, captures the diverse ways Iranian women engage public spaces to assert their rights and initiate social transformation—ranging from organizing protests and demonstrations to the creation of public art such as murals and graffiti (Mehan, 2024b).

By framing our analysis within these theoretical and methodological considerations, this paper contributes to a deeper understanding of the complexities surrounding informal feminist placemaking in Iran. It not only sheds light on the specific strategies employed by Iranian women to navigate and reshape their urban environments but also situates these actions within a broader discourse on gender, space, and resistance. Through this exploration, the aim is to reveal the nuanced interplay between space, gender, and activism, offering insights into the transformative potential of informal placemaking practices under authoritarian regimes.

## 2 Methodological notes

This research adopts a qualitative framework, utilizing a mixed-methods strategy to explore the nuances of informal feminist placemaking within the Iranian context. Our investigation began with an extensive literature review, focusing on academic books, peer-reviewed articles, and monographs dedicated to feminist and informal placemaking practices in the global south. This literature, spanning both English and Farsi/Persian languages, was sourced from the library database at Texas Tech University Huckabee College of Architecture, ensuring a diverse and comprehensive theoretical foundation for our study.

The subsequent phase of our research centered on primary data collection, conducted during the Spring semester of 2023 within the course “Community Design and Development Resources” that have been taught in Spring 2023 at Texas Tech University Huckabee College of Architecture. This phase highlighted the complexity of defining “feminist urban space,” underscoring the subjective nature of feminist placemaking. Our analysis acknowledges that these spaces are shaped by the values and priorities of their inhabitants, making any universal definition elusive. Instead, our goal is to explore the processes that illuminate the dynamic relationships between gender, public space, religion, and state authority, aiming to identify commonalities and divergences across different conceptualizations of feminist placemaking processes globally.

A significant challenge faced in this research is the issue of censorship in Iran, particularly concerning data related to the ‘Women, Life, Freedom’ Movement and other grassroots initiatives. The imperative for participant confidentiality necessitated the anonymization of contributions and the omission of direct quotations. Such censorship may have influenced participants’ openness in sharing their experiences and insights. Despite these constraints, our study offers valuable perspectives on the diverse expressions of feminist placemaking in Iran, carefully navigating the complexities without seeking to define a singular model of feminist urbanism.

Our findings provide a thematic overview that lays the groundwork for future empirical research. While acknowledging the context-specific nature of our insights—which may limit their applicability across different cultural or regional settings—we believe they contribute significantly to the understanding of feminist placemaking, particularly in the global south. This study, therefore, serves as an initial step toward a more detailed exploration of the evolution of protest movements, their objectives, and forms, within and beyond the Iranian context.

## 3 Informal placemaking practices and women’s participation in the public sphere

Placemaking, as conceptualized within the Western context, has evolved from the foundational works of scholars like Relph (1976), Tuan (1977), and Whyte (1980), who have explored the thematic and experiential aspects of human connections to places. Whyte’s seminal studies on public plazas in New York highlight the importance of these spaces in urban life (Whyte, 1980). In the Anglo-Western paradigm, placemaking is seen as a socio-political and geo-specific community engagement process aimed at creating ‘positive’ public space outcomes through the reimagining and redevelopment of social, cultural, and spatial settings such as plazas, squares, and promenades (Beza, 2016; Kozlowski et al., 2020). This process is characterized by a collaborative dialog among the community, stakeholders, and the government to achieve spatial planning outcomes that are negotiated and positively perceived (Strydom et al., 2018; Mehan and Stuckemeyer, 2023a, 2023b), embodying principles of “direct citizen participation” and “community engagement” (Kalandides, 2018). An underlying and fixed assumption embedded within the placemaking process in the global north is that the community equally has the right and capacity to participate (Khasraghi and Mehan, 2023).

To ensure community participation, based on Tamayo and Cruz Guzman’s definitions, four conditions must be met:

1. that […] individuals have the freedom to make public use of their reason […];
2. that […] individuals are autonomous, with their capacity to participate;
3. that state power is put under public judgment; and
4. This judgment is the fruit of consensus (Tamayo and Cruz-Guzman, 2008, 49)

These conditions suggest that placemaking can influence the inclusive production of public spaces. However, spatial, and economic barriers, often resulting from market-based decision-making, can limit citizen participation (Irazábal, 2008).

The concept of “informality” plays a crucial role in understanding placemaking’s broader implications. Massey (1994) describes a place’s uniqueness as stemming from a “constellation of social relations” that converge within a specific locale, often outside government regulation. This informality is pivotal in feminist placemaking, a nuanced approach where women assert their presence in physical spaces, challenging and transforming traditional gender norms and structures through their engagement in unregulated social and economic activities.

Feminist placemaking represents a distinct form of women’s participation in the public sphere, extending beyond political, economic, cultural, and social interactions to include the transformation of physical spaces. This engagement allows women to navigate and reshape their environments, offering alternative models of participation and influence. The article delves deeper into how these practices resist and restructure power dynamics and social injustices, contributing to a more inclusive understanding of placemaking.

By examining feminist placemaking through the lens of informality, this revised section aims to provide a clearer, more coherent narrative that aligns with the reviewer’s suggestions. It enhances the explanation of key concepts with examples, restructures the manuscript for better thematic organization, broadens the engagement with existing literature, and deepens the analysis of power dynamics and social practices in shaping public spaces.

## 4 Informal placemaking practices in the global south

The concept of Informal Feminist Placemaking in the Global South is pivotal in understanding how the built environment can sustain or challenge existing power dynamics. This framework, which emphasizes the transformative practices, processes, and urban activist-led initiatives, amplifies the perspectives of marginalized groups, focusing particularly on women and non-binary individuals (Dyck, 2005; Serag, 2016). Drawing on Rendell’s (2011) notion of ‘critical spatial practices,’ this approach advocates for resistance and creative activism against the social order of global corporate capitalism. Rendell (2011) articulates the need for action and resistance to address the urgent challenges of our time, such as environmental crises and political conflicts, through creativity and social critique.

In the Global South, where power imbalances are pronounced, Informal Feminist Placemaking seeks to question and confront these prevailing structures while promoting alternative norms and ideals (Varış Husar et al., 2023). Gender, as a fundamental category in cultural organization, often manifests in ways that favor men over women, underscoring the need for an ontological reframing toward a more experimental, performative, and ethical orientation (Reckitt and Phelan, 2001; Gibson-Graham et al., 2013).

This approach is inherently community-driven, allowing marginalized groups, especially women and non-binary individuals, to assert control over their urban environments (Mehan, 2020; Mehan et al., 2023). This assertion of control can take various forms, including the creation of community gardens, the establishment of pop-up parks, and the execution of street art projects, which not only claim space but also foster a sense of community and belonging (Bezner-Kerr and Zuhroh, 2017). Such interventions underscore the right of every community member, particularly those in marginalized positions, to shape their environment and highlight the importance of safe and inclusive public spaces in achieving equitable and just cities (Zuhroh and Davids, 2018).

The persistence of gender segregation in the Global South, deeply embedded in historical, social, and economic systems controlled by dominant institutions and individuals, necessitates innovative strategies for community empowerment. Through mechanisms like community land trusts and co-operatives, Informal Feminist Placemaking provides sustainable and long-term avenues for communities to influence the development of their neighborhoods (Bezner-Kerr and Zuhroh, 2017). This methodology is crucial in addressing the unique challenges faced by marginalized communities in the Global South, contributing significantly to the creation of more equitable and just urban environments.

## 5 Gendered state-led placemaking agency in contemporary Iran

The feminist movement in contemporary Iran has undergone significant transformations influenced by several factors, such as the Islamic Revolution of 1979, the ongoing struggle for democracy, and the evolving roles of women in modern Iranian society. The Islamic Republic has actively promoted a traditional and patriarchal model of gender roles, resulting in limitations on women’s educational and employment opportunities and discouraging their participation in political spheres. This imposition of gender norms has challenged conventional notions of private ownership and Western understandings of the public sphere (Nawratek and Mehan, 2020). Within the Islamic conceptualization of public space, the veil assumes a central role in the gendered dynamics of navigating both public and private domains.

The veil, as a symbolic marker, embodies a form of visibility that draws attention to cultural domains encompassing dress codes and urban esthetics, while simultaneously regulating the gaze of Muslim men within public spaces. Women’s experiences of public and private spaces in Islamic societies are shaped by the need for personal space, territorial control, and adherence to Islamic norms of modesty. These experiences are influenced by the socio-cultural expectations surrounding the veil, emphasizing the complexities of women’s engagement with public spaces (Deep and Harb, 2013; Zamani and Mehan, 2019; Ali et al., 2020). The Islamic discourse on the distinction between the private and public spheres is grounded in economic and environmental values, emphasizing the granting of private ownership as a reward for individual work outcomes (Arjmand, 2017).

The new regime was deeply conservative, patriarchal, and sought to roll back many women’s gains in the previous decades. This significantly reduced women’s participation in the public sphere, including in the workforce and political life. Especially, Ayatollah Khomeini’s call for women in Iran to wear the long black garment that covers the whole body, leaving only the face exposed, has been defined as the pinpoint in returning Iranian women to a shrouded life (Jaynes, 1979). Ayatollah Khomeini referred to the chador as the “Flag of the revolution” (Sciolino, 2003). This revolutionary “chador (means veiling)” slogan was part of the state’s gender segregation plan, which scrutinized women’s and men’s bodily presentation in urban spaces. As the bearers of Islamic morality and ethical subjects, men and women should be separated in public spaces (Shahrokni, 2020). To prevent the mixing of unrelated men and women and to prevent the “sin,” women had to pass through checkpoints installed at the entrances of universities, shopping malls, airports, theaters, and government buildings (Sciolino, 1992).

Regarding women’s access to public space based on the state’s policies, gender segregation provides a theoretical lens to explain the paradoxical contradictions of Iranian society over the past four decades. To create the “Islamic City,” a new set of spatial imperatives have been created to divide places along gender lines and mark them with visual screens such as walls, signs, and fences (Shahrokni, 2020).

In Lila Abu-Lughod’s work on resistance titled “The Romance of Resistance,” she explores how the concept of “romance as resistance” motivated feminists and journalists to uncover the hidden aspects of Tehran (Abu-Lughod, 1990). By focusing on the experiences of Bedouin women in Egypt’s Western Desert, Abu-Lughod highlights their defiance of sexual segregation and oppressive practices (Abu-Lughod, 1990). In Tehran, this concept may inspire individuals to challenge societal norms and power structures, revealing the hidden corners of the city and shedding light on marginalized voices. Abu-Lughod’s work serves as a valuable framework to understand how resistance and exploration can uncover the untold stories of Tehran and promote social change. To describe the new socio-political topography of Tehran, describes it as “the city of lies” where “passionate uprisings” had been stirred up in the “underground world” where segregation was breached (Khatib, 2014; Navai, 2014). Also, in the public life of Metropolitan cities like Tehran, by wearing colorful coats and scarves instead of “chadors,” women were “conquering enclosed public spaces” (Amir-Ebrahimi, 2006). The state’s never-ending prohibitive policies were often contested. On March 8, 1979, several thousand “bare-headed” women “dressed in blue jeans and jackets” marched out from Tehran University, raising their fists against mandatory veiling, “refusing religion-defined womanhood” (Moghissi, 2009). However, in response, the gender-segregated spaces suggested by conservatives rapidly expanded. Following the conceptualization of “pastoral power” and Iris Marion Young’s “logic of masculinist protection,” the state presents itself as the protector of the “fragile” women’s bodies that are the products of its earlier policies (Young, 2003; Foucault, 2007).

The gender segregation regime of the 1980s can be characterized by exclusion, closure, and prohibition, while the regime in the 2000s shifted toward inclusion, opening, and provision (Shahrokni, 2020). By distinguishing between exclusion, closure, and prohibition in the 1980s and inclusion, opening, and provision in contemporary times, Shahrokhi highlights the evolving strategies employed by state actors to shape gendered state-led placemaking. This transformation is exemplified by the introduction of women-only transportation options such as buses, metro cabins, and taxis, which aimed to create dedicated spaces for women (Banakar and Payvar, 2015). Rather than focusing solely on spaciousness, the emphasis was on separating men and women to align with Islamic principles.

The state’s reinforcement of gender segregation extended beyond transportation. Women-only parks, cafes, restaurants, city complexes (referred to as “Shahrbanu” in Farsi), internet cafes, schools, and universities witnessed significant expansion, particularly with the rise of conservative influences (Vakil, 2011). It is important to note, however, that these women-only spaces sometimes evolved into “alternative or distinct public spheres” due to the practices of the individuals who frequented them (Habermas, 1989; Fraser, 1990).

## 6 Informal revolutionary feminist placemaking

Feminist Revolutionary Placemaking is an approach to designing, creating, and transforming public spaces that aim to promote gender equality and asserting the agencies of the marginalized groups, specifically women. This approach challenges traditional patriarchal norms and societal expectations imposed on women and other marginalized groups and seeks to create inclusive and safe, spaces for all (Mehan, 2015). Over the past four decades, Iranian women have used the “power of presence” as an approach to the feminist movement to resist being pushed out of the public domain. This has been demonstrated through the social media campaign “My Stealthy Freedom,” which began on Facebook in 2014 and used the hashtag #whitewednesdays. Every Wednesday, images of Iranian women with their hair uncovered and hijabs (veils) held aloft appear on social media (Mehan and Rossi, 2019; Mehan, 2022). In the current decade, Iranian women have been engaging in different waves of activism. They are protesting their lack of bodily autonomy and the compulsory wearing of hijabs by climbing on platforms and benches in public spaces. These protests were sparked by an Iranian woman who stood on a utility box in Tehran’s Revolution Street on December 28, 2017. The young protesters, known as “daughters of the revolution,” wave white scarves tied to poles to symbolize their protest. However, it is critical to highlight that the struggle is not just about the hijab itself but rather the more significant issue of gender politics and state control over women’s bodies. This is evident in the reenactment of the initial protest by other women, who have come to be known as the “Girls of Revolution Street” on social media (Hoodfar, 2018).

**Figure 1:**
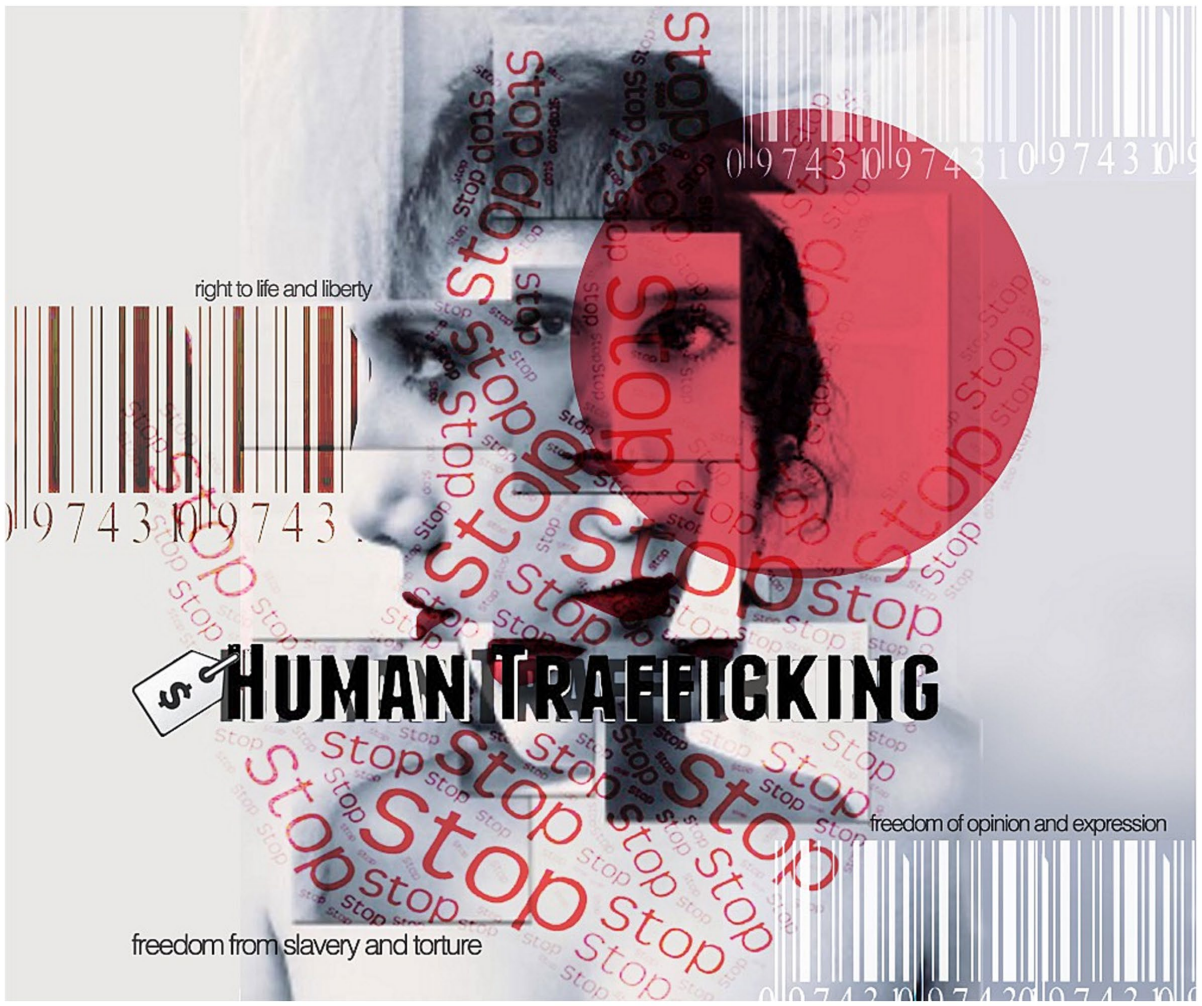
This image serves as a poignant reminder of the role feminist placemaking plays in combatting human trafficking. It underscores the need for safe, inclusive spaces that uphold the dignity and rights of all women, free from exploitation and violence. Source: Karla D. Hernandez and Tahseen Reza Anika, 2023.

Feminist revolutionary placemaking is not limited to Iran but can be seen in various places worldwide. These marches sought to reclaim public spaces, specifically at night, which were traditionally considered unsafe for women. The marches allowed women to assert their right to move freely in public spaces without fear of harassment or violence (see Figure 1).

Addressing counterarguments enriches the discourse around Feminist Revolutionary Placemaking. Critics often point to the societal and governmental resistance that such movements face, questioning their efficacy in effecting long-term systemic change. Concerns about backlash, including legal actions and social stigma, are also prevalent, potentially deterring participation in these feminist efforts (Hoodfar, 2018). However, acknowledging these challenges underscores the resilience and strategic adaptability of feminist movements. The digital dimension of activism, for example, has extended the reach and impact of these movements beyond geographical and societal limitations, fostering a global network of solidarity and support. This adaptability suggests that Feminist Revolutionary Placemaking is not only persisting in the face of adversity but also evolving to become a more inclusive and powerful agent of social change.

In addition to physical spaces, feminist revolutionary placemaking also applies to digital spaces (Tappert et al., 2024). Online platforms such as social media have become powerful tools for women to organize and mobilize and to claim their right to exist and to be heard in the digital public sphere (Mostafavi and Mehan, 2023). The #MeToo movement, which started in 2017, is an example of how digital spaces can be used for feminist revolutionary placemaking. Through hashtags and social media, women worldwide could share their stories of sexual assault and speak out against the systemic patriarchal structures that have allowed these abuses to continue (Mehan, 2023c, 2023d, 2023e).

Furthermore, data on the global spread and influence of the #MeToo movement could offer insights into its transformative impact across different countries and sectors. Visuals and statistics on participation in feminist protests and digital campaigns illuminate the growing momentum behind these movements, highlighting not only the scale of engagement but also the tangible shifts in public discourse and policy reforms that have been stimulated by such activism. Through this approach, the narrative around Feminist Revolutionary Placemaking gains depth, showcasing the movement’s tangible impacts on societal attitudes toward gender equality and the visibility of feminist activism. This discussion not only addresses the critiques and challenges faced by the movement but also highlights the significant achievements and ongoing evolution of feminist placemaking strategies in promoting inclusivity and justice in both the physical and digital realms.

Feminist revolutionary placemaking is a powerful tool for promoting gender equality and asserting the agencies of the marginalized groups, specifically women. Whether in physical or digital spaces, feminist revolutionary placemaking is a way for women and other marginalized groups to assert their agency and claim their right to exist and to be heard in the public sphere. As an example of feminist revolutionary placemaking, the next section will focus on the “Woman, Life, Freedom” movement, led by Iranian women who challenge traditional societal norms and government policies that restrict their rights and limit their agency in public spaces.

## 7 ‘Woman, life, freedom’ movement

The phrase “Women, life, freedom” has gained prominence as a slogan for the feminist movement in Iran, which was sparked by the death of 22-year-old Mahsa (Jina) Amini in September 2022. Amini passed away while in the custody of the morality police for alleged violation of Iran’s strict hijab laws. Her death mobilized social media, and videos of women defying the government by removing their headscarves, cutting their hair, and protesting spread quickly. As a result, the government shut down the internet to try to quell public demonstrations.

The movement primarily demands the abolishment of laws that discriminate against women, including the obligatory hijab law that enforces women to cover their hair in public. Furthermore, the movement advocates for the cessation of patriarchal attitudes and practices, such as forced marriages and domestic violence, which serve to undermine women’s rights and freedom. The “Woman, Life, Freedom” movement has witnessed substantial growth and support via social media and digital platforms, acting as primary means for activists to disseminate information, mobilize supporters, and organize protests.

The movement has gained traction and support through social media and online platforms, which have allowed activists to share information, mobilize, and organize protests and campaigns. The movement began as a social media campaign, with Iranian women posting pictures of themselves without wearing the mandatory headscarf, or hijab, in public spaces. The campaign quickly gained traction, with thousands of women participating and sharing their stories of harassment and discrimination for not wearing the hijab. Despite the government’s attempts to silence and repress the movement, it has continued to grow and gain momentum. Reflecting on Fielding-Smith, during the revolution, background, religious affiliation, political affiliation, regional affiliation, and ethnicity were not considered (Fielding-Smith, 2011).

The reference to Fielding-Smith (2011) in the context of the “Woman, Life, Freedom” movement serves to underscore the unifying nature of the protests, transcending individual differences such as background, religious affiliation, political views, and ethnicity. Fielding-Smith’s analysis suggests that during revolutionary movements, traditional divisions are often set aside in favor of common goals, illustrating the formation of nonhierarchical and horizontal relationships among participants. This concept aligns with the idea of rhizomes proposed by Deleuze and Guattari (1994), where movements grow and spread through interconnected yet decentralized networks, embodying a collective force without central leadership (Deleuze and Guattari, 1994, 110). In the case of the “Woman, Life, Freedom” movement, this framework helps explain how diverse groups of Iranian women and allies come together, leveraging both digital and physical spaces to challenge authoritarian constraints and advocate for gender equality.

This process of becoming involves “people to come” who are missing or lacking in the actual world and have the chance to invent themselves by resisting what is intolerable in the present. This act of asserting one’s presence in the public sphere through vocal protest is a crucial aspect of street-level resistance, which can escalate from small acts of insubordination to larger insurrections against authoritarian power (Elkin, 1985; Phillips, 1998; Ahmed, 2017). In this context, a poignant example of such collective action occurred at one of Tehran’s Art Universities. In solidarity with the “Woman, Life, Freedom” movement, female students, forbidden to sing in public under Iran’s Islamic regime, performed a well-known revolutionary chant. This performance, which took place in the campus’s main building—where women’s performances have been proscribed for years—garnered participation from both male and female students. Spectators were invited to join the singing, a symbolic act of defiance against the imposed restrictions.

**Figure 2:**
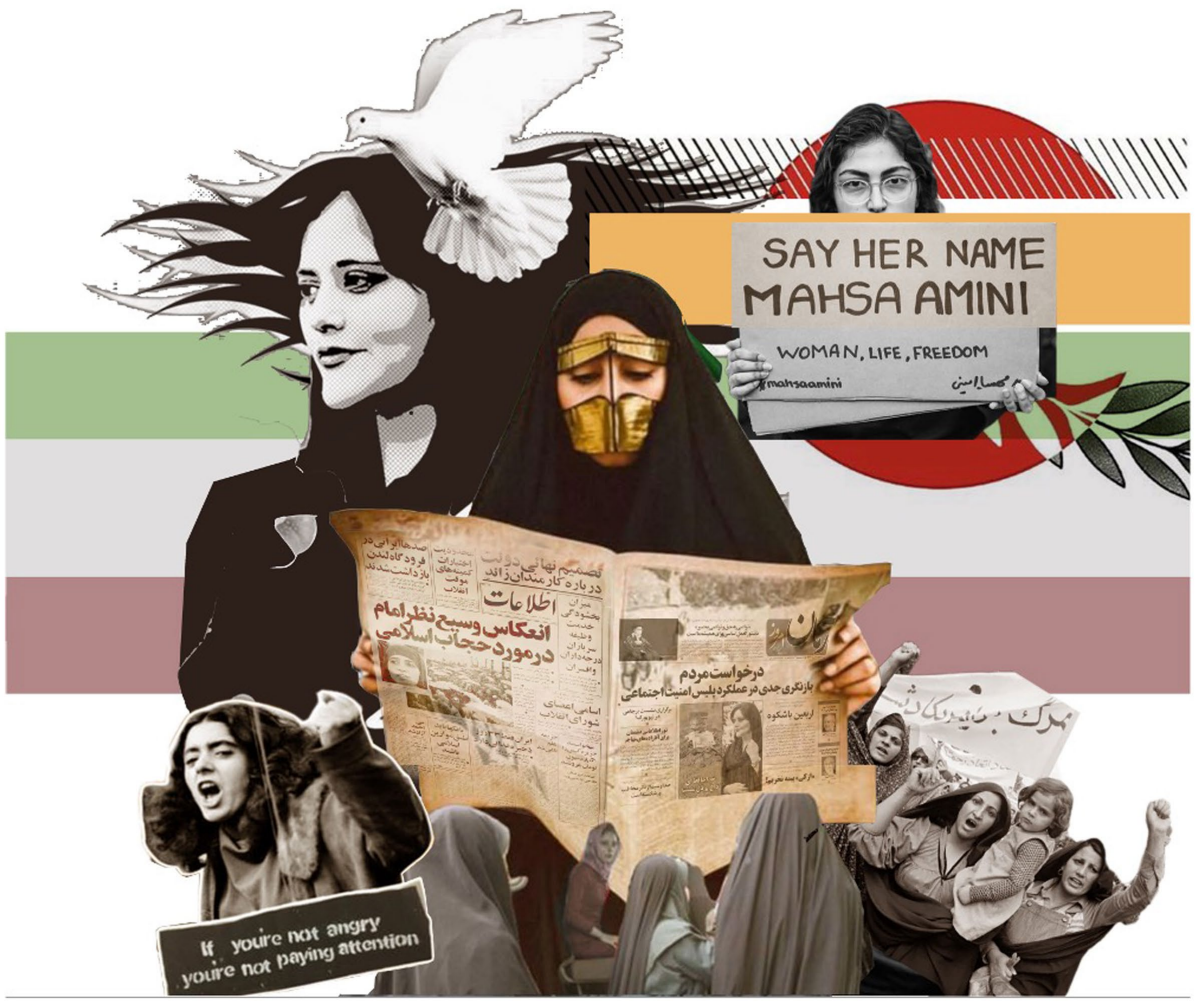
A collage of defiance and peace: a juxtaposition of women’s activism and symbolism in ‘Woman, Life, Freedom’ Movement with layered images of protest, hashtags, cultural elements, and a call for freedom. Sources: Karla D. Hernandez and Tahseen Reza Anika, 2023.

**Figure 3:**
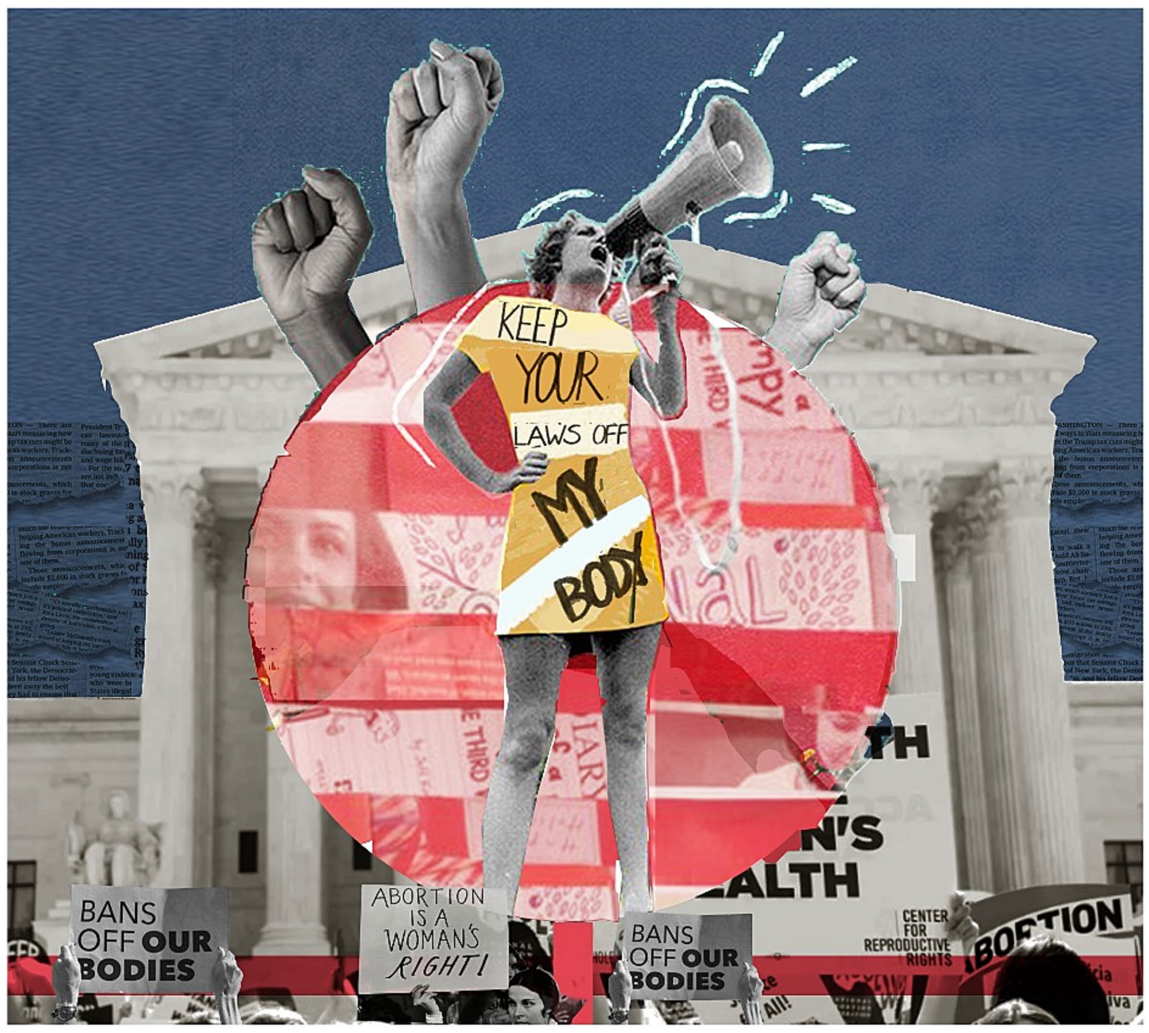
A powerful montage advocating bodily autonomy: the iconic Supreme Court backdrop amplifies the fervor of protestors defending reproductive rights, encapsulated by a vibrant red overlay symbolizing urgency and solidarity. Sources: Karla D. Hernandez and Tahseen Reza Anika, 2023.

The movement’s resistance extends beyond the digital realm to physical public spaces, where women engage in civil disobedience acts like publicly removing their hijabs and holding placards bearing the movement’s slogan. These acts are shaping a new narrative for women in Iran and transforming public spaces’ perception and use. Despite the state’s punitive responses—manifested in harsh repression, arrests, and imprisonment—the movement continues to evolve. Women leverage social media, digital art, graffiti, street art, and other creative expressions to claim public spaces, thereby asserting their right to self-expression and autonomy, and further challenging the state’s repressive mechanisms (see Figures 2, 3).

The “Woman, Life, Freedom” movement represents a critical juncture in feminist activism within Iran, propelled by the tragic death of Mahsa (Jina) Amini and marked by significant acts of defiance against systemic oppression. Through a combination of digital activism and physical protests, Iranian women and their global allies have showcased remarkable courage and resilience, challenging not only specific laws like the mandatory hijab but also broader issues of gender discrimination, forced marriages, and domestic violence.

The movement has harnessed the power of social media to circumvent state censorship, amplify voices, and organize protests, illustrating a significant shift toward digital feminist activism. Despite facing severe state repression, the “Woman, Life, Freedom” movement continues to evolve, with women at the forefront of claiming their rights and reshaping the public discourse on gender equality in Iran and beyond. This grassroots, informal placemaking has not only raised awareness about women’s rights issues in Iran but has also contributed to a global dialog on gender equality, showcasing the interconnectedness of feminist struggles worldwide.

## 8 Concluding notes

The quest for women’s rights in Iran is a tenacious struggle that mandates the ongoing efforts of not only women but also men, and the government. This study delves into the key role of informal revolutionary feminist placemaking in Iran, centering on the “Women, Life, Freedom” Movement. It accentuates how this form of placemaking empowers activists to contest traditional gender norms by seizing public spaces as platforms for activism and resistance. As discussed in the main body of this research, informal placemaking often arises from grassroots movements, providing space for innovation and spontaneity, yet it may lack sustained resources and formal recognition. Formal placemaking, by contrast, typically benefits from organizational support and funding but may be subject to bureaucratic delays or be less responsive to localized needs. Both types can interact, with informal initiatives becoming formalized over time, and formal programs incorporating informal, community-led activities (Mehan & Mostafavi, 2023). State-led placemaking can have extensive resources and a broad reach but may not address specific local needs or empower local communities. Conversely, bottom-up initiatives, often being community-led, have the advantage of being highly responsive to local needs, but might struggle with limited resources or capacity, and potential clashes with state-level policies (Mehan, 2023f).

The commonalities among these diverse forms of placemaking lie in their shared goal of reshaping public space to address gender equality, while their differences stem from the variance in the origin of initiatives, resources, and approaches to community engagement. Challenges span across resource constraints, state suppression, and social stigma, while opportunities arise in the form of mobilizing public support, fostering innovation, and driving systemic change.

Revolutionary feminist placemaking, as embodied by the “Women, Life, Freedom” Movement, is a potent force in challenging traditional norms and patriarchal structures, even amidst the challenges of state suppression and social stigma. Iranian state, to suppress the movement, employs a wide array of mechanisms to suppress the “Woman, Life, Freedom” movement, necessitating critically exploring these tactics. Among the notable techniques is internet censorship, a manifestation of state power aimed at disrupting the digital momentum of the movement and obstructing inter-activist communication. However, the sustained mobilization and organization of protests, despite such impediments, underscores the resilience of the activists and challenges the efficacy of the state’s digital control. Furthermore, the state apparatus—evident in law enforcement and discriminatory legislation such as the mandatory hijab law—functions as a tool of social control. Nevertheless, the public defiance exhibited by activists disrupts the intended impact of these oppressive laws, simultaneously symbolizing resistance and revealing cracks in the state’s power structure.

The state’s reliance on surveillance and intimidation strategies also merits attention. While designed to discourage activist participation, the movement’s continued growth in the face of such tactics attests to the potency of feminist defiance. The state’s use of punitive measures, such as arrests and imprisonment, paradoxically enhances the movement’s visibility, attracting domestic and international attention and inadvertently bolstering support for the cause. In addition, the state’s endorsement of restrictive cultural norms limits women’s participation in public spaces. In a counteractive stance, activists engage in placemaking—public performances, art creation—to challenge these norms and reassert women’s claim to public spaces.

A critical analysis of state suppression tactics reveals a dichotomy—while these tactics demonstrate the extensive reach of state power, they are simultaneously met with activist resilience that challenges and undermines this power. This resistance, primarily through the lens of placemaking, offers a compelling counter-narrative to state control and highlights the transformative potential of the feminist movement. The solidarity-action frame became dominant among activists fighting for freedom as they pushed for equality among Iranian women involved in the movement. This led to the creation of inclusive alliances driven by a collective desire for equality. In this interpretation, informal feminist placemaking in the context of non-democratic societies can be viewed as essential manifestations of the constitutive dimension of politics, specifically for subaltern groups and minorities who experience oppression and violence, which are not mobilizing for a specific end but to assert their presence in the public sphere and to speak out against injustices. Finally, it’s important to note the critical role of feminist placemaking in building a more inclusive society in Iran. Despite daunting challenges including state censorship, social stigma, and resource scarcity, the inspiring efforts of activists showcase the potential to establish empowering spaces for women. Hence, the steadfast commitment to organize, protest, and raise awareness is a testament to the resilience and transformative potential of the Iranian feminist movement. Future studies should delve deeper into the nuances of these placemaking practices, examining their unique and shared impacts on the struggle for women’s rights in Iran and beyond.

## Author contributions

AM: Conceptualization, Data curation, Formal analysis, Funding acquisition, Investigation, Methodology, Project administration, Resources, Supervision, Validation, Visualization, Writing – original draft.
